# Association of the gut microbiota with coronary artery disease and myocardial infarction: A Mendelian randomization study

**DOI:** 10.3389/fgene.2023.1158293

**Published:** 2023-04-11

**Authors:** Dan Wang, Xiaoyan Chen, Zhen Li, Ying Luo

**Affiliations:** Department of Geriatric Medicine, Xiangya Hospital, Central South University, Changsha, China

**Keywords:** Mendelian randomization, gut microbiota, coronary artery disease, myocardial infarction, causality

## Abstract

**Background:** Previous studies have indicated that the gut microbiota (GM) is associated with coronary artery disease (CAD), but the causality of these associations remains unestablished due to confounding factors and reverse causality. We conducted Mendelian randomization study (MR) to determine the causal effect of the specific bacterial taxa on CAD/myocardial infarction (MI) and identify the mediating factors involved.

**Methods:** Two-sample MR, multivariable MR (MVMR) and mediation analysis were performed. Inverse-variance weighting (IVW) was the main method used to analyze causality, and sensitivity analysis was used to verify the reliability of the study. Causal estimates from CARDIoGRAMplusC4D and FinnGen databases were combined using the meta-analysis method, and repeated validation was conducted based on the UK Biobank (UKB) database. Confounders that may affect the causal estimates were corrected by MVMP and the potential mediation effects were investigated by using mediation analysis.

**Results:** The study suggested that increased abundance of the RuminococcusUCG010 genus leads to a lower risk of CAD (OR, 0.88; 95% CI, 0.78, 1.00; *p* = 2.88 × 10^−2^) and MI (OR, 0.88; 95% CI, 0.79, 0.97; *p* = 1.08 × 10^−2^), with consistent results in both meta-analysis (CAD: OR, 0.86; 95% CI, 0.78, 0.96; *p* = 4.71 × 10^−3^; MI: OR, 0.82; 95% CI, 0.73, 0.92; *p* = 8.25 × 10^−4^) and repeated analysis of the UKB dataset (CAD: OR, 0.99; 95% CI, 0.99, 1.00, *p* = 2.53 × 10^−4^; MI: OR, 0.99; 95% CI, 0.99, 1.00, *p* = 1.85 × 10–11). Based on multiple databases, T2DM was proved as a mediating factor in the causal effect of RuminococcusUCG010 and CAD/MI, with an average mediation effect proportion of 20% on CAD and 17% on MI, respectively.

**Conclusion:** This MR study provided suggestive genetic evidence that the higher the RuminococcusUCG010 abundance is, the lower the risk of CAD and MI, with T2DM playing a mediating effect. This genus may become a novel target in strategies for treating and preventing CAD and MI.

## 1 Introduction

Cardiometabolic diseases refer to a clinical syndrome primarily caused by metabolic disorders, with atherosclerotic cardiovascular disease as the main manifestation, except for structural cardiovascular pathologies, arrhythmias and cardiomyopathies (Zhu, 2021). There is an explicit causal relationship between metabolic abnormalities and cardiovascular diseases. Intervention in metabolic disorders can effectively improve prognosis (Saxon et al., 2020). Coronary artery disease (CAD), the main type of cardiometabolic disease, is the leading cause of morbidity and mortality worldwide, accounting for approximately nine million deaths in 2017, and the prevalence of CAD is projected to increase to more than 1,845 cases per 100,000 individuals, with an upper confidence estimate of 1,917 cases per 100,000 individuals, by 2030 (Mozaffarian et al., 2015; Khan et al., 2020). Cardiometabolic risk factors, such as type 2 diabetes, dyslipidaemia, obesity, and insulin resistance, are related to an increased risk of CAD. In recent decades, treatment targeting these traditional risk factors has significantly reduced cardiovascular mortality. However, despite recent advances, residual risk of CVD remains (Kotseva et al., 2016; Kotseva et al., 2017). As the prevalence of CAD increases globally under the current control measures, efficient new strategies are necessary to target CAD and modifiable risk factors. The gastrointestinal tract is where nutrients the body requires are digested and absorbed and where the greatest number and diversity of microbiota are found; the gut microbiota (GM) is a key regulator of metabolism and exhibits relative stability and resilience over time (Rinninella et al., 2019; Afzaal et al., 2022). Accumulating evidence has revealed that the GM and metabolites are involved in human metabolism, immunity and inflammation processes and are involved in the development and progression of CAD (Kazemian et al., 2020; Novakovic et al., 2020; Xu et al., 2020).

Trimethylamine-N-oxide (TMAO) is an intestinal microbiota-dependent metabolite from dietary choline, betaine, and L-carnitine that is metabolized by trimethylamine lyases to form trimethylamine (TMA) and catalyzed by the action of flavin monooxygenases in the liver (Bennett et al., 2013). TMAO inhibits reverse cholesterol transport, which augments cholesterol accumulation within macrophages and promotes foam cell formation to form atherosclerotic plaques. In addition, TMAO has a proinflammatory effect (Yamashita et al., 2021). Studies have demonstrated a critical role of TMAO in pro-atherogenesis in germ-free mice (Wang et al., 2011). In several large independent clinical cohorts, plasma TMAO levels were shown to be associated with the risk of CAD morbidity and long-term mortality and thus predictors of cardiovascular risk (Tang et al., 2013; Wang et al., 2014). However, in the MR study, there is no direct causal relationship between TMAO and CAD, but a suggestive association of genetically increased choline with a higher risk of T2DM (Jia et al., 2019) and the same effect of TMAO and carnitine with systolic BP (Wang et al., 2022). Butyrate is a short-chain fatty acid (SCFA) produced by the metabolism of intestinal microflora, which has been proved to be a protective metabolite for CAD in contrast to TAMO (Hu et al., 2022). However, there is a lack of MR studies. In a comparative study of CAD patients and controls, patients exhibited characteristic changes in the composition and abundance of the GM. A relative reduction in *Bacteroides* and Prevotella and enrichment in *Streptococcus*, *Escherichia*, and Collinsella were observed in patients (Karlsson et al., 2012; Jie et al., 2017). Stephan J’s study confirmed the presence of bacteria in atherosclerotic lesions in 2006 with a high overall bacterial diversity of more than 50 different species (Ott et al., 2006). Later, in Omry Koren’s and Ju Seung Kwun’s study, the bacterial taxa isolated from atherosclerotic plaques were found to exist in the gut of the same individuals and the amount of bacterial DNA correlated with a amount of leukocytes in the atherosclerotic plaque, which indicates that the amount of bacteria contributes to the inflammatory status of the atherosclerotic plaque (Koren et al., 2011; Kwun et al., 2020). These observations suggest that the bacteria in plaques are homologous to the GM. Therefore, the GM potentially promotes plaque formation. Based on these findings, more research has been performed to investigate the mechanisms and potential therapeutic targets of the GM in CAD. Jill Gregory et al. found that atherosclerosis susceptibility could be transferred to ApoE null mice through transplantation of a high/low TMAO-producing strain of the GM (Gregory et al., 2015). A novel Bowman-Birk type major trypsin inhibitor (FMB-BBTI) was shown to markedly restrain atherosclerosis progression by remodeling the structure of the GM, as verified in a mouse model (Shan et al., 2022). In a randomized controlled study of 44 CAD patients, probiotic supplementation (*Lactobacillus* rhamnosus GG, LGG) had beneficial effects on metabolic endotoxemia, and mega inflammation in patients. The levels of biomarkers of inflammation and metabolic endotoxemia, such as IL-1 beta and hs-CRP, decreased significantly in the LGG group compared to the placebo group (Moludi et al., 2021). Increasing evidence indicates that the GM may represent a novel therapeutic target for CAD, and GM-based CAD therapies include gut flora transplantation, probiotic supplementation of specific taxa, and other modulation of the GM (Warmbrunn et al., 2020).

However, current studies on the correlation between the GM and CAD still have limitations. Small study samples may lead to biased results and underestimate some important predictors. Whether the mutual association is causal remains undetermined according to traditional epidemiological studies due to residual confounding factors and reverse causation bias. In addition, randomized controlled trials, as the gold standard for inferring a causal association, have ethical restrictions and are impractical. Mendelian randomization (MR), however, is a method for assessing causal associations by using genetic variants as instrumental variables (IVs). MR can prevent the possible effects of residual confounding and reverse causality because genetic variants are assorted randomly and defined at conception (Smith and Ebrahim, 2003; Zoccali et al., 2006; Verduijn et al., 2010). Therefore, we conducted a two-sample MR study to explore the causal associations of the GM with CAD/myocardial infarction (MI) and identify specific causal bacterial taxa as potential prevention and treatment targets. Confounding factors that may affect the causal estimates were corrected by multivariable MR (MVMR) and the potential mediation effects were investigated by using mediation analysis.

## 2 Materials and methods

### 2.1 Study design

**FIGURE 1:**
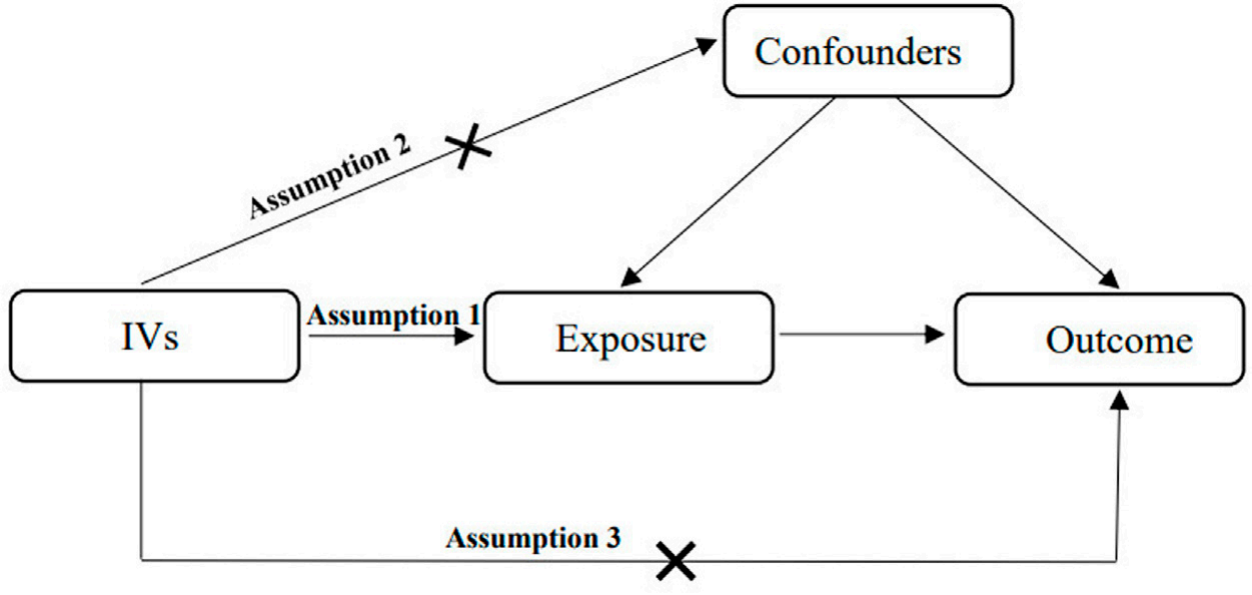
The critical assumptions of MR analysis.

**FIGURE 2:**
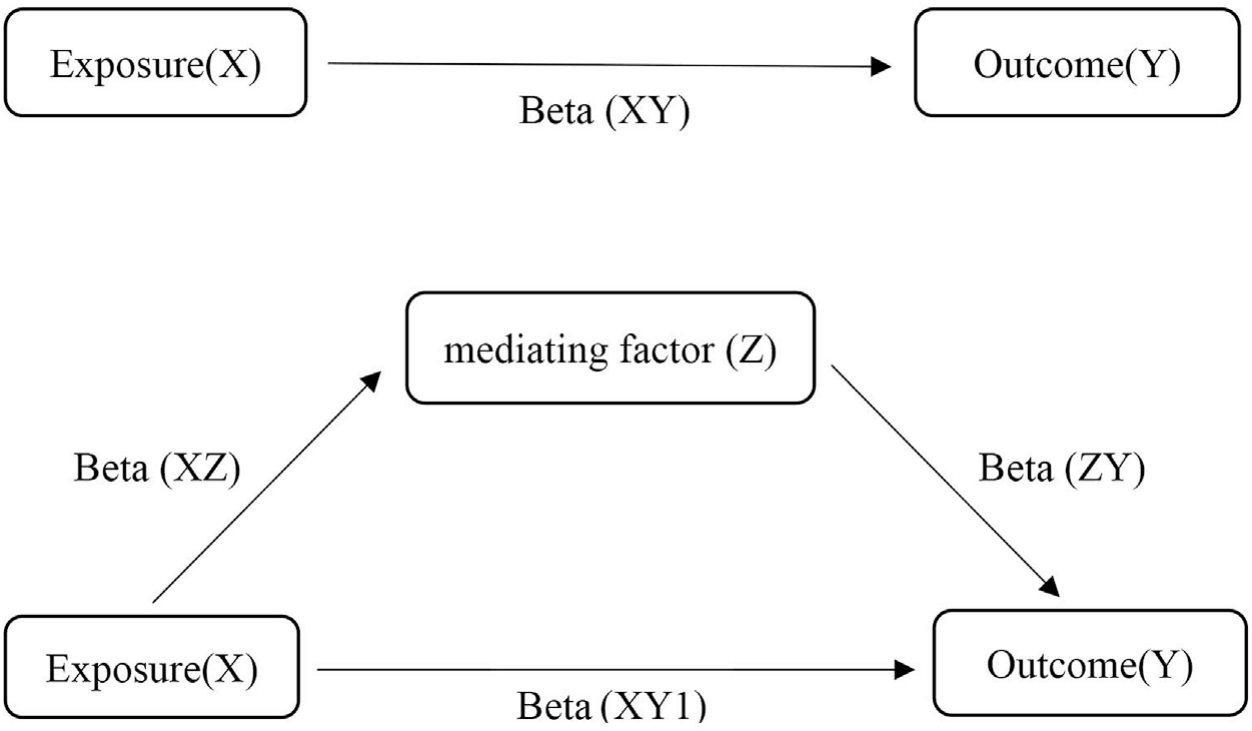
The assumptions of Mediation analysis.

MR is used to identify and estimate a causal effect using genetic instrumental variables. Two-sample MR assesses two independent populations (same ethnicity) using single nucleotide polymorphisms (SNPs) as IVs (Lawlor, 2016). It is generally not associated with confounders of conventional observational studies, such as postnatal environmental factors, socioeconomic status, behavior and reverse causality, because the genotypes of germline genetic variation are defined at conception (Smith and Ebrahim, 2003; Smith et al., 2007). The MR approach follows Mendel’s law of inheritance, which is considered analogous to randomized controlled trials. There are three critical assumptions of MR analysis (Figure 1). First, the relevance assumption is that IVs are robustly associated with exposure. Second, the independence assumption is that IVs are absolutely independent of any confounders that may affect the exposure and outcomes. Third, the exclusion restriction assumption is that the association between the IVs and outcomes is exclusively dominated by exposure instead of alternative mechanisms (Emdin et al., 2017). In this two-sample MR study, we made use of publicly accessible datasets from large-sample genome-wide association studies (GWASs) for both GM (as a risk factor) and CAD/MI (as a disease outcome) to estimate the causal inferences. Sensitivity analysis was used to verify the reliability of the causal association results. As a protective metabolite of CAD/MI, butyrate may play a potential mediating effect between GM and CAD/MI. Therefore, we conducted synchronously MR studies of butyrate and CAD/MI. MVMR is an extension of univariate MR, which takes into account the pleiotropy of genetic variants that the same genetic variation may dominate multiple phenotypes (Burgess and Thompson, 2015). The causal effect of exposure on outcomes was direct or indirect depending on the role of confounding factors that as a risk for outcome may have overlapping effect with exposure. MVMP can be used to verify and correct the confounders. Mediation analysis is based on the framework of two-sample MR to assess the mediating effect of significant confounders in the causal association that exposure X leads to a change in mediator Z, which in turn results in a change in outcome Y (Figure 2) (Burgess et al., 2014). Our MR study followed the MR-STROBE guidance to strengthen the Reporting of Observational Studies in Epidemiology using MR (Skrivankova et al., 2021).

### 2.2 Data sources

In the two-sample MR, the SNPs that were used as IVs were extracted from the MiBioGen consortium, the largest and latest GM-GWAS, involving 18,473 participants in 25 cohorts dominated by European descent. The GWAS generated 122,110 variant sites from 211 taxa at 5 levels (from genus to phylum) (Kurilshikov et al., 2021). We obtained summary-level data for CAD/MI from the CARDIoGRAMplusC4D and FinnGen databases. The CARDIoGRAMplusC4D 1000 Genomes-based GWAS is a meta-analysis of CAD-GWASs involving 60,801 CAD (43,676 MI cases) cases and 123,504 controls, nearly 77% individuals with European ancestry (Nikpay et al., 2015). The FinnGen consortium is a large genomic database of half a million Finns, established in 2017 (https://www.finngen.fi/en), including 25,707 CAD cases (defined by the International Classification of Diseases (ICD)−8, code 410 or 4110; ICD−9, code 410 or 4110 and ICD−10, code I20, I21 or I22) and 15,787 MI cases (defined by ICD−8, code 410, ICD−9, code 410 and ICD−10, code I21 or I22) (FinnGen, 2022). Additionally, we validated the analyses using UK Biobank (UKB) database, a general population prospective cohort study recruiting approximately 500,000 participants from the United Kingdom. The purpose of that study was to assess the genetic and lifestyle-related risk factors for various chronic diseases, and the database included 10,157 CAD cases and 7,018 MI cases (Sudlow et al., 2015). Plasma butyrate levels are challenging to measure in current assays, so it is difficult to obtain the direct genetic estimation of butyrate in a large population cohort. PWY-502 is a microbial pathway involved in 4-aminobutanoate (GABA) degradation, the abundance of which was used as a proxy for butyrate production in the intestine. The summary-level data of PWY-502 is based on the Life Lines DEEP (LL-DEEP) queue, that is a subset of the largest Lifelines biobank consisting of 1,539 individuals from Northern Netherlands. (Bonder et al., 2016; Sanna et al., 2019). In the MVMP and Mediation analysis, the summary-level data of type 2 diabetes (T2DM) were extracted from FinnGen and a meta-analysis of three GWAS data: Diabetes Genetics Replication and Meta-analysis (DIAGRAM), Genetic Epidemiology Research on Aging (GERA) and UKB (Xue et al., 2018). Essential hypertension was obtained from FinnGen and UKB. Global Lipid Genetics Consortium (GLGC) as a source of genetic data for Low-density lipoprotein cholesterol (LDL-C), involved in 188,578 European-ancestry individuals (Willer et al., 2013).

### 2.3 Genetic instrumental variables

In our study, SNPs associated with the GM at 5 taxonomic levels (phylum, class, order, family, genus, and species) were screened from the MiBioGen study as IVs. A range of filtering criteria were used to select qualified IVs. First, SNPs associated with the GM were extracted at a *p*-value less than the locus-wide significance level (1 × 10^−5^). Second, Linkage disequilibrium (LD) refers to the non-random association of alleles at different loci that means the probability of two alleles located on the same chromosome simultaneously is greater than the probability of random distribution in the population. To ensure the independence of each SNP from distinct bacterial taxa, the clumping process was performed with reference to the European samples from the 1000 Genomes Project, with an R2 < 0.001 and a window size = 10,000 k. (Sved and Hill, 2018). Third, SNPs with a minor allele frequency (MAF) less than 0.01 were removed to avoid statistical bias. Fourth, the SNPs located on sex chromosome 23 were removed. Fifth, SNPs associated with potential confounders were excluded across PhenoScanner V2 (http://www.phenoscanner.medschl.cam.ac.uk/) to ensure the second core assumption of MR. Sixth, if selected SNPs were not available in the outcome dataset, proxy SNPs with a high linkage disequilibrium (LD) (r2>0.80) were identified by using SNiPa (http://snipa.helmholtz muenchen. de/snipa3/) as an alternative. Seventh, the MR Steiger filtering test was conducted to identify the third key hypothesis. This approach examined whether exposure is directional to the causality of the outcome and eliminated SNPs with reverse causality. Eighth, the F statistic was defined as $F=R^{2}\left(n-k-1\right)/k\left(1-R2\right)$ to evaluate the bias of the weak instrument, where R2 represents the proportion of variation interpreted by selected SNPs. SNPs with an F- value less than 10 were deemed weak instruments (Burgess and Thompson, 2011). Ninth, palindromic SNPs were eliminated in the harmonizing process to ensure that the same SNP on the exposure and outcome corresponded to the same allele. SNPs that met the above criteria were included in the study as was effect allele (EA), MAF, effect size (β), standard error (SE), *p*-value, and other corresponding information.

### 2.4 Statistical analysis

#### 2.4.1 Two-sample MR

Inverse-variance weighting (IVW), MR Egger, weighted median estimator (WME), and simple median analyses were performed to infer the causal inferences. IVW was the main analysis method, and the intercept was constrained to zero. The meta-analysis of IVW combined the Wald estimates for each SNP (Burgess et al., 2013). The point estimates of IVW were equivalent to a weighted linear regression of SNP-outcome associations on SNP-exposure associations (Bowden et al., 2017). Fixed and random effects IVW approaches were used. MR‒Egger analysis was performed by a simple modification to the weighted linear regression of IVW. MR‒Egger analysis differs from IVW in that it does not set the intercept term to zero, and the term is estimated to assess pleiotropy as part of the analysis (Bowden et al., 2015). If the intercept term is close to zero or not statistically significant, then the slope term in MR‒Egger regression represents the estimated causal effect of exposure on the outcome, which is close to the IVW estimate; otherwise, it indicates the existence of horizontal pleiotropy. However, in contrast to the traditional methods for estimating the causal effects of MR, MR‒Egger analysis violates the InSIDE hypothesis that the pleiotropic effects of the genetic variants in the analysis are uncorrelated with the associations of the variants with the risk factor, which have been shown to lead to increased bias and type 1 error rate inflation (Burgess and Thompson., 2017; Lin et al., 2022). Moreover, the results of MR‒Egger regression are susceptible to outlying variants, which can lead to reversal of the horizontal pleiotropy test and causal effect estimates (Burgess and Thompson., 2017). The simple median estimator is the median ratio estimate, which is equivalent to a weighted median estimator with equal weights. When less than 50% of the genetic variants are invalid, the simple median can provide a consistent estimate of the causal effect (Han, 2008). The WME estimate is the median of a weighted empirical density function of the ratio estimates, which can provide a consistent estimation of the causal effect even when more than 50% of the weight comes from invalid IVs. WME is more accurate than the simple median when individual estimates differ greatly in accuracy (Bowden et al., 2016).

The sensitivity analysis in this study was conducted in three aspects. We tested for multiplicity of validity by MR Egger and Mendelian randomized multiplicity residuals and outliers (MR-PRESSO). As described above, when the MR Egger intercept term was not close to zero or statistically significant, it indicates the presence of horizontal multiplicity (Bowden et al., 2015). When the proportion of multiplicity IVs is less than 50%, MR-PRESSO can be used as an effective method for identifying horizontal multiplicity outliers in MR analyses and contains three main functions. First, the MR-PRESSO global test is used to test for the presence of horizontal pleiotropy. Second, the MR-PRESSO outlier test is used to identify and remove pleiotropic outliers and recalculate relatively unbiased estimates. Third, the MR-PRESSO distortion test provides significant differences in the causal estimates before and after correction for outliers (Verbanck et al., 2018). The heterogeneity of the SNPs included was tested by Cochran’s Q test and I2 statistics in the IVW model. Cochran’s Q test can be used to test the heterogeneity of each independent study. If the results were statistically significant, significant heterogeneity was demonstrated. The I2 statistic is calculated as I2 = [(Q–df)/Q] x 100%, reflecting the proportion of the heterogeneous component in the total variance (Higgins and Thompson., 2002). Mild heterogeneity was considered when the I2 statistic ranged from 0% to 25%, moderate heterogeneity when it ranged from 25% to 50%, and high heterogeneity when it was greater than 50%. The leave-one-out sensitivity test identifies strongly influential SNPs by removing individual SNPs one by one to re-estimate the total effect with IVW and determine the degree of influence of single SNPs on the association estimates by the stability of the effect estimates. All statistical analyses were conducted in R version 4.0.5 using the Two-Sample MR (https://github.com/MRCIEU/TwoSampleMR), MR-PRESSO (https://github.com/rondolab/MR-PRESSO), Forestplot (https://rdrr.io/cran/forestplot/f/vignettes/forestplot.Rmd). Power calculations were performed by the online tool mRnd (https://shiny.cnsgenomics.com/mRnd/) with the minimum effect >80% power. Sample size, Type-I error rate, proportion of cases, the odds ratios (ORs) and R2 were included in the calculation as the main parameters. In multiple testing, Bonferroni-corrected *p* values of <0.05/2 were considered statistically significant.

#### 2.4.2 Multivariable MR

Based on the two-sample MR, confounders with the same causal effect as GM were included in the MVMR. IVs extracted in MVMP is a combination of multiple exposures associated with at least one of the GM and confounders. IVW was also the main analysis method. The analysis was conducted using the packages Two-Sample MR and Mendelian Randomization (https://cran.r-project.org/package=MendelianRandomization) in R.

#### 2.4.3 Mediation analysis

The mediating effect is calculated as $Beta=Beta\left(XZ\right)\times Beta\left(ZY\right)$ , $R=\frac{Beta}{Beta\left(XY\right)}\times 100\%$ . Beta (XZ), Beat (XY) respectively represents the effect of exposure on mediators and outcome. Beta (ZY) indicates the effect of mediators on the outcome after correcting for the effect of exposure. R is the proportion of mediating effect in the total effect. The effect of exposure on outcome after correction for confounders is considered as direct effect.

## 3 Results

### 3.1 The two-sample MR

**FIGURE 3:**
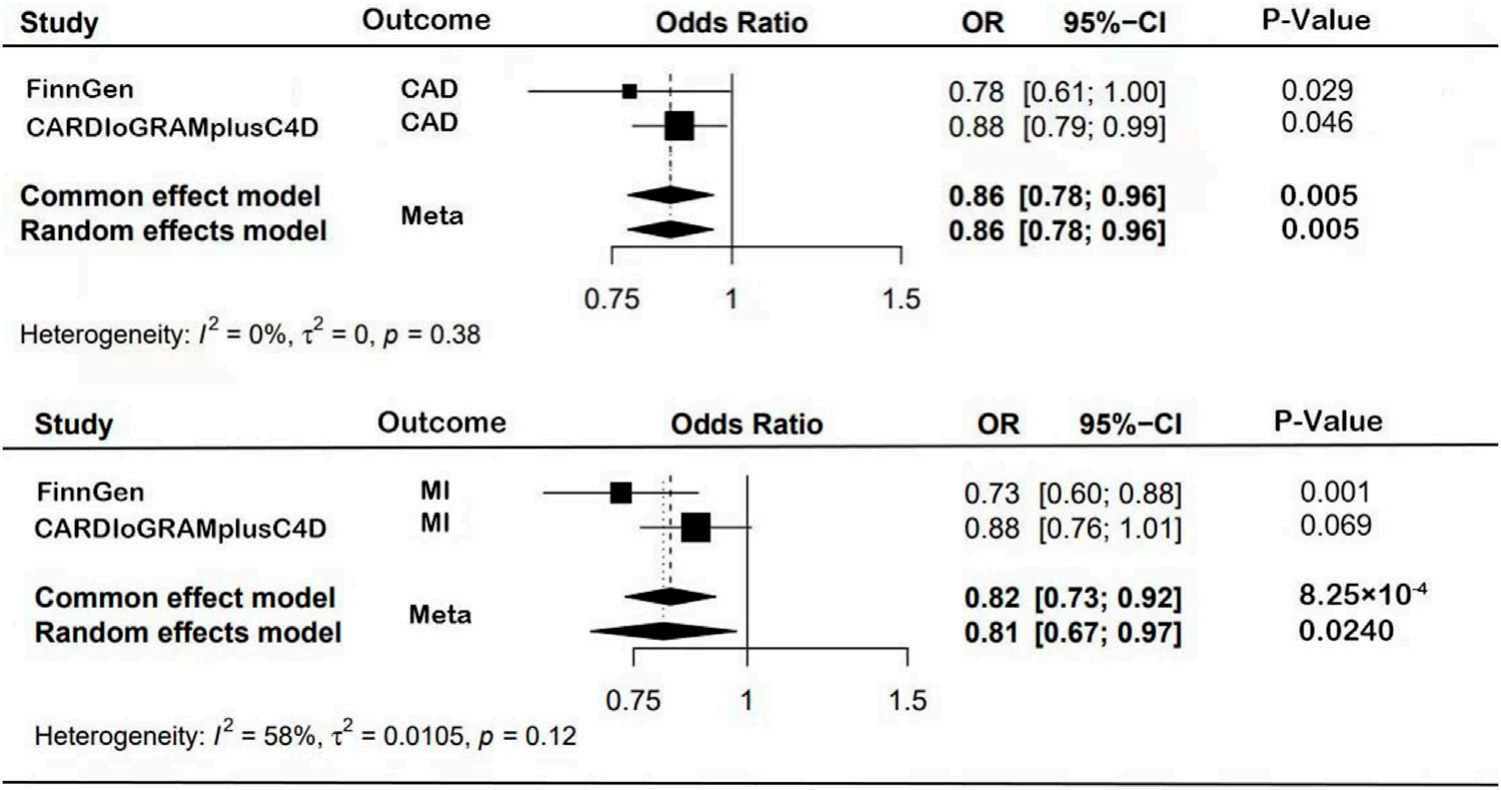
Forest plot results from MR study.

The MR-STROBE guidance is shown in Supplementary Table S1. Table 1 provides details of the studies and samples used in the MR analyses. The majority of all study participants were adults of European ancestry. Bacterial taxa were analyzed in the MR study at five levels, including 9 phyla, 16 classes, 20 orders, 35 families and 131 genera. A distinct taxon was defined as a feature. Detailed information is shown in Supplementary Table S2. RuminococcusUCG010 was the only genus that maintained consistent positive results in multiple databases and meta-analyses (Table 2; Figure 3). Twenty SNPs closely related to the genus RuminococcusUCG010 were screened from the MBG. database (*p* < 1 × 10^−5^). Twelve no independent SNPs (“rs34668774", "rs686403", "rs10281901", "rs591790", "rs686246", "rs592858", "rs8056635", "rs73218806", "rs886017", "rs2773827", "rs3088274", "rs7661237") were removed from the LD test (r2<0.001, Kb = 10,000), and 2 palindromic SNPs (rs35506912, rs7935775) were removed in the harmonization process. The remaining 6 SNPs were all present in the outcome database as IVs. These six SNPs were not found to be associated with other confounders in the PhenoScanner, and all had MAFs greater than 0.01 and F values well above 10. Supplementary Table S3 provides information about the SNPs, including chromosome, effect allele, non-effect allele, effect allele frequency, *etc.* The results of the MR Steiger filtering test are presented in Supplementary Table S4. The six IVs explained more genetic variation in the GM than CAD or MI, indicating causality in the expected direction. These SNPs could explain 10.06% of the phenotypic variance in the genus RuminococcusUCG010 (Supplementary Table S5).

**TABLE 1:** Detailed information of data sources for analyses.

Data source	Phenotype	Sample size	Cases	Population	Covariates adjusted in GWAS
MiBioGen	gut	18,340	—	78% European	Age、BMI、alpha-diversity
CARDIoGRAMplusC4D	CAD	184,305	60,801	77% European	Not reported
	MI	171,875	43,676		
FinnGen	CAD	260,405	25,707	European	Age, sex, and up to 20 genetic principal components
	MI	238,338	15,787		
UK Biobank	CAD	361,194	10,157	European	Age, sex, and 10 genetic principal components
	MI	361,194	7,018		
GRAMplusGERAplusUKB	T2DM	659,316	62,892	European	Age, sex
FinnGen	T2DM	16,380,440	—	European	Age, sex, and up to 20 genetic principal components
UK Biobank	Essential hypertension	337,199	500	European	Age, sex, and 10 genetic principal components
FinnGen	Essential hypertension	205,694	42,857	European	Age, sex, and up to 20 genetic principal components
GLGC	LDL-C	188,578	—	European	Age, sex

GM, gut microbiota; CAD, coronary artery disease; MI, myocardial infarction; CARDIoGRAMplusC4D, Coronary Artery Disease Genome−Wide Replication and Meta−Analysis plus the Coronary Artery Disease Genetics.

**TABLE 2:** Associations of genetically predicted RuminococcusUCG010 with CAD and MI.

Data source	Outcome	SNPs	Method	OR	95% CI	*p*−Value
CARDIoGRAMplusC4D	CAD	6	IVW (fixed effects)	0.88	0.78,1.00	0.052
			IVW (random) effects)	0.88	0.79,0.99	0.029
			WME	0.89	0.75,1.04	0.149
			MR Egger	0.88	0.57,1.35	0.592
			Simple median	0.88	0.75,1.02	0.097
CARDIoGRAMplusC4D	MI	6	IVW (fixed effects)	0.88	0.76,1.01	0.069
			IVW (random) effects)	0.88	0.79,0.97	0.011
			WME	0.92	0.77,1.10	0.381
			MR Egger	1.06	0.66,1.69	0.820
			Simple median	0.87	0.73,1.04	0.127
FinnGen	CAD	6	IVW (fixed effects)	0.78	0.67,0.92	0.003
			IVW (random effects)	0.78	0.61,1.00	0.046
			WME	0.77	0.62,0.97	0.026
			MR Egger	0.63	0.31,1.27	0.264
			Simple median	0.78	0.62,0.97	0.029
FinnGen	MI	6	IVW (fixed effects)	0.73	0.60,0.88	0.001
			IVW (random) effects)	0.73	0.61,0.86	2.75E-04
			WME	0.74	0.57,0.97	0.026
			MR Egger	0.68	0.40,1.16	0.233
			Simple median	0.76	0.58,1.00	0.043
UKB	CAD	6	IVW (fixed effects)	0.99	0.99,1.00	0.164
			IVW (random) effects)	0.99	0.99,1.00	2.35E-04
			WME	0.99	0.99,1.00	0.040
			MR Egger	0.99	0.97,1.00	0.235
			Simple median	0.99	0.99,1.00	0.038
UKB	MI	6	IVW (fixed effects)	0.99	0.99,1.00	0.006
			IVW (random) effects)	0.99	0.99,1.00	1.85E-11
			WME	1.00	0.99,1.00	0.066
			MR Egger	0.99	0.98,1.00	0.219
			Simple median	1.00	0.99,1.00	0.051

CAD, coronary artery disease; MI, myocardial infarction; CARDIoGRAMplusC4D, Coronary Artery Disease Genome−Wide Rep-lication and Meta−Analysis plus the Coronary Artery Disease Genetics; IVW, inverse variance weighted; WME, weighted median estimator; MR−Egger, Mendelian randomization−Egger.

**FIGURE 4:**
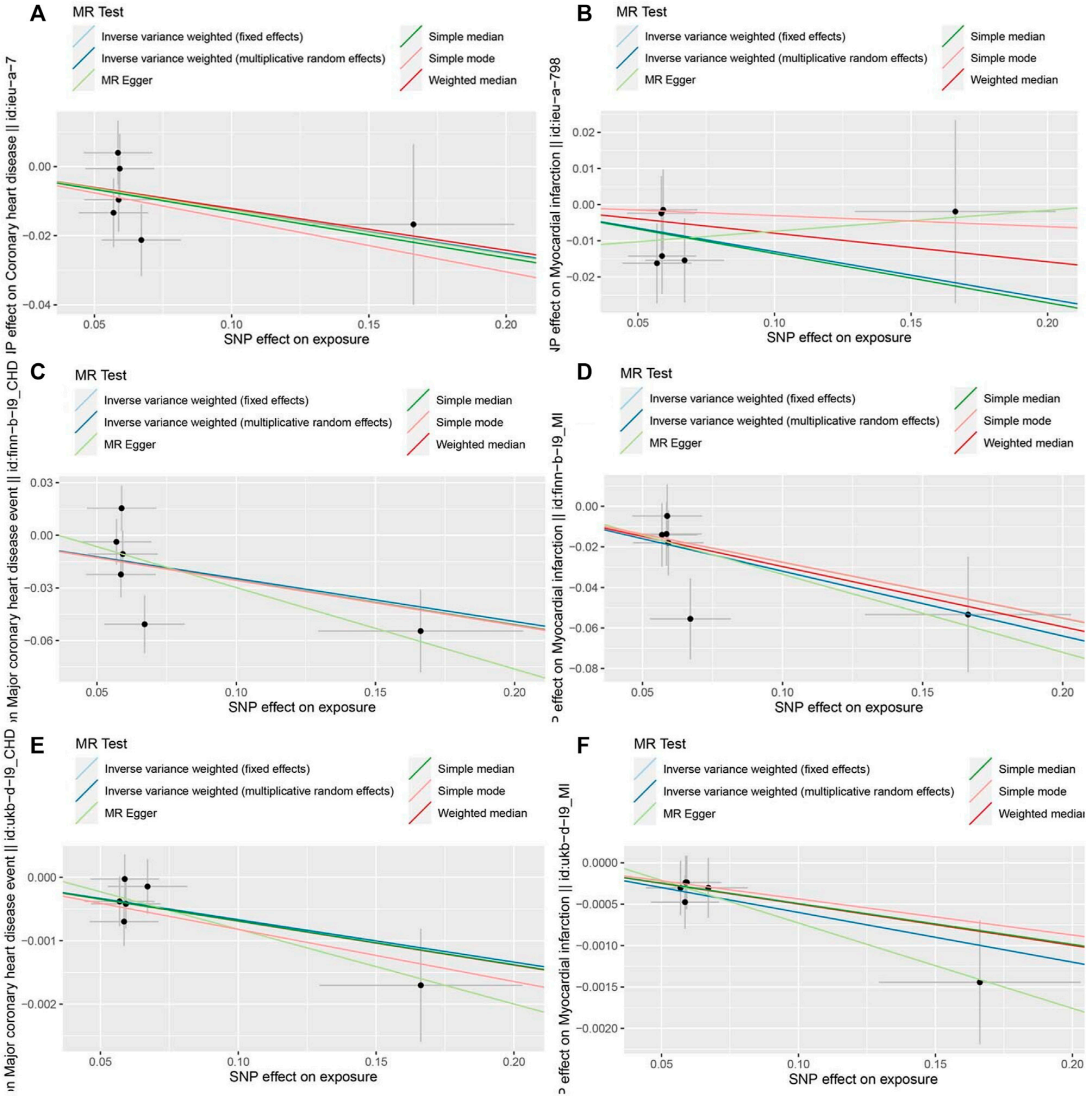
Scatter plots of the MR estimates for the association of RuminococcusUCG010 on CAD/MI. **(A)**. RuminococcusUCG010-CAD based on CARDIoGRAMplusC4D. **(B)**. RuminococcusUCG010-MI based on CARDIoGRAMplusC4D. **(C)**. RuminococcusUCG010-CAD based on FinnGen. **(D)**. RuminococcusUCG010-MI based on FinnGen. **(E)**. RuminococcusUCG010-CAD based on UKB. **(F)**. RuminococcusUCG010-MI based on UKB.

The MR estimates from different methods of assessing the causal effect of RuminococcusUCG010 with CAD/MI are shown in Table 2. In this study, according to IVW as the primary causal assessment method, the risk of CAD or MI was reduced by 12% when the abundance of RuminococcusUCG010 doubled the standard deviation in the random effect IVW model based on the CARDIoGRAMplusC4D dataset (CAD: OR, 0.88; 95% CI, 0.78, 1.00; *p* = 2.88 × 10^−2^; MI: OR, 0.88; 95% CI, 0.79, 0.97; *p* = 1.08 × 10^−2^). All analyses based on the FinnGen dataset were positive, except for MR‒Egger analysis. The meta-analysis of CARDIoGRAMPlusC4D and FinnGen data maintained consistent protection (CAD: OR, 0.86; 95% CI, 0.78, 0.96; *p* = 4.71 × 10^−3^; MI: OR, 0.82; 95% CI, 0.73, 0.92; *p* = 8.25 × 10^−4^; Figure 3). To further verify the consistency of the association of the abundance of RuminococcusUCG010 with CAD or MI, the analysis was repeated based on the UKB. The above causal effects persisted in the MR analysis (CAD: random-effects IVW model: OR, 0.99; 95% CI, 0.99, 1.00, *p* = 2.53 × 10^−4^; Weighted median: OR, 0.99; 95% CI, 0.99, 1.00, *p* = 3.97 × 10^−2^; Simple median analysis: OR, 0.99; 95% CI, 0.99, 1.00, *p* = 3.81 × 10^−2^; MI: Fixed-effects IVW model: OR, 0.99; 95% CI, 0.99, 1.00, *p* = 5.63 × 10^−3^; random-effects IVW model: OR, 0.99; 95% CI, 0.99, 1.00, *p* = 1.85 × 10^–11^). Nevertheless, caution is needed here, as several associations did not reach the Bonferroni-corrected *p*-value of 0.0125. The scatter plots for each outcome were shown in Figure 4.

**FIGURE 5:**
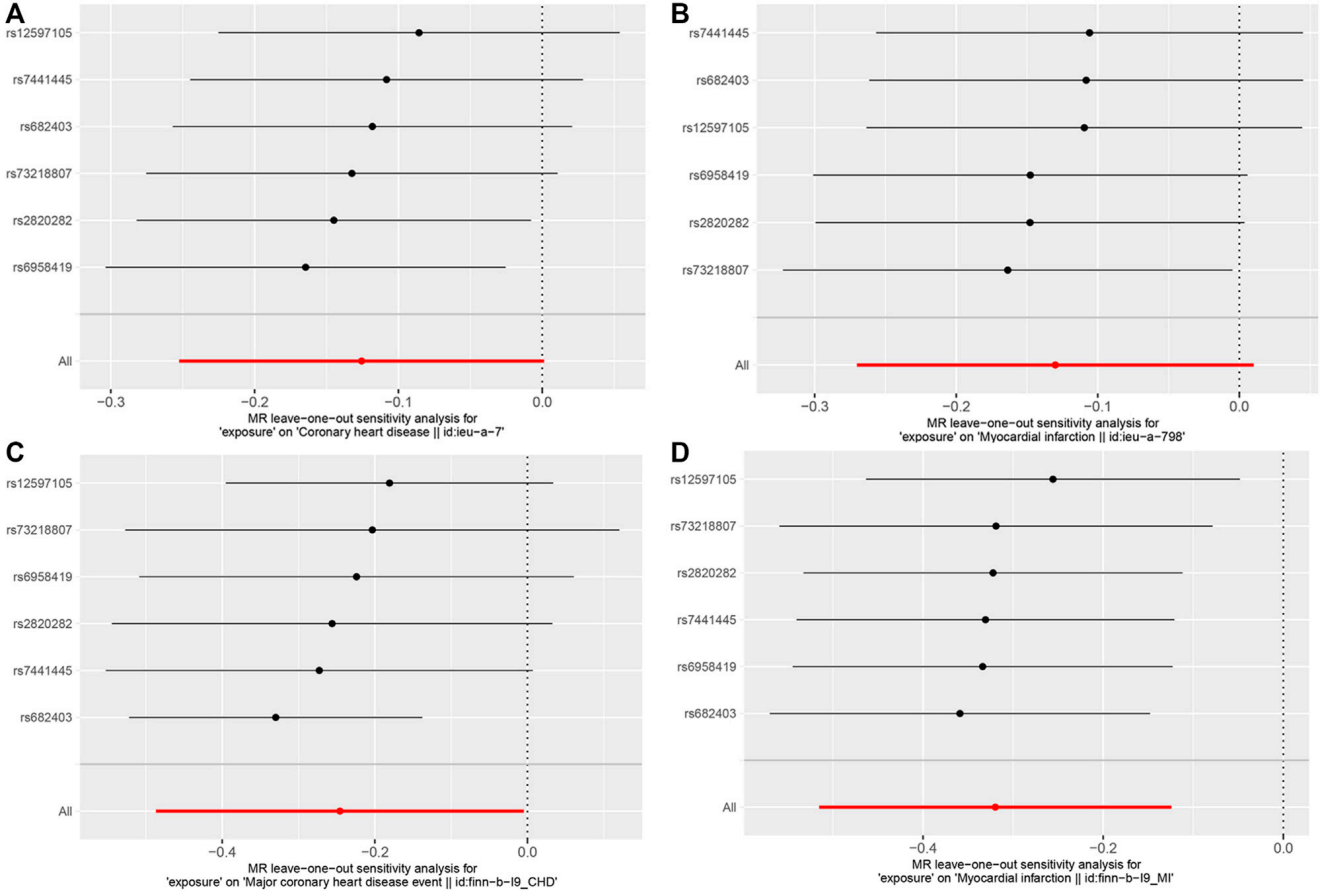
Results of the leave-one-out analysis. **(A)**. The leave-one-out analysis for RuminococcusUCG010 on CAD based on CARDIoGRAMplusC4D. **(B)**. The leave-one-out analysis for RuminococcusUCG010 on MI based on CARDIoGRAMplusC4D. **(C)**. The leave-one-out analysis for RuminococcusUCG010 on CAD based on FinnGen. **(D)**. The leave-one-out analysis for RuminococcusUCG010 on MI based on FinnGen.

In the Cochran Q and I2 tests, there was a certain heterogeneity in the SNPs associated with the GM and FinnGen (P Cochran’s Q = 0.05, I2 = 54%), with no heterogeneity in the rest (Table 3). There was no horizontal pleiotropy detected in either the MR‒Egger intercept P or MR-PRESSO global test P tests (P intercept >0.0125; P global test >0.0125, Table 3). In addition, no outlier SNP to be corrected was found in the MR−PRESSO outlier test, and no potential SNP driving causality was found in the leave-one-out analysis (Figure 5). This study had over 80% statistical power for detecting the associations between the GM and CAD/MI based on the CARDIoGRAMplusC4D and FinnGen studies, respectively (Supplementary Table S5). However, the statistical power based on the UKB was restrictive, because of the shortage of CAD/MI cases or the deficiency variance in the GM explained by the selected IVs.

**TABLE 3:** Evaluation of heterogeneity and directional pleiotropy using different methods on RuminococcusUCG010 with CAD/MI.

Data source	Outcome	Heterogeneity		Pleiotropy	
		I2 (%)	Cochran’s Q p	MR−Egger intercept p	MR−PRESSO global test p
CARDIoGRAMplusC4D	CAD	0	0.558	0.992	0.610
	MI	0	0.770	0.456	0.789
FinnGen	CAD	54%	0.050	0.547	0.125
	MI	0	0.567	0.811	0.689
UK Biobank	CAD	0	0.782	0.560	0.780
	MI	0	0.974	0.557	0.980

CARDIoGRAMplusC4D, Coronary Artery Disease Genome−Wide Replication and Meta−analysis plus the Coronary Artery Dis-ease Genetics; CAD, coronary artery disease; MI, myocardial infarction; MR−Egger, Mendelian randomization−Egger; MR−PRESSO, MR−pleiotropy residual sum and outlier.

### 3.2 MVMR study and mediation analysis

A suggestively association was observed for a higher genetically predicted abundance of RuminococcusUCG010 with T2DM risk based on both the DIAGRAMplusGERAplusUKB (OR: 0.81, 95% CI: 0.71, 0.93, *p* = 1.75 × 10^−3^) and FinnGen (OR: 0.79, 95% CI: 0.69, 0.90, *p* = 3.27 × 10^−4^). There was no obvious causal effect between RuminococcusUCG010 and LDL-C, essential hypertension. The results of MR estimates are shown in Supplementary Table S6. There was no heterogeneity in the Cochran Q and I2 tests (Supplementary Table S7).

Nine genetic predictors for PWY-5022 was screened out as IVs to calculate association (Supplementary Table S8). We did not detect any causal relationships between the abundance of PWY-502 and CAD/MI (Supplementary Table S9). There was a certain heterogeneity between PWY-5022 and CAD based on the FinnGen (P Cochran’s Q = 0.05, I2 = 36%), with no heterogeneity in the rest (Supplementary Table S10).

T2DM and RuminococcusUCG010 was included as exposures for MVMR. The association of genetically predicted RuminococcusUCG010 with CAD/MI disappeared after adjusting for T2DM in the MVMR. There was no causal effect of RuminococcusUCG010 on CAD or MI in the correction model (Table 4). The mediation effect of T2DM was presented in Table 5 with an average proportion of 20% on CAD and 17% on MI, respectively.

**TABLE 4:** Causal effect of RuminococcusUCG010 on CAD/MI, conditioned on T2DM.

T2DM					
		DIAGRAMplusGERAplusUKB		FinnGen	
		OR (95% CI)	p	OR (95% CI)	p
CAD	CARDIoGRAMplusC4D	0.77, 1.28	0.94	0.69, 1.57	0.84
	FinnGen	0.73, 1.31	0.87	0.70, 1.55	0.84
MI	CARDIoGRAMplusC4D	0.82, 1.34	0.68	0.65, 1.55	0.99
	FinnGen	0.77, 1.46	0.70	0.69, 1.67	0.75

CAD, coronary artery disease; MI, myocardial infarction; T2DM, Type 2 diabetes; CARDIoGRAMplusC4D, Coronary Artery Disease Genome-Wide Replication and Meta-analysis plus the Coronary Artery Disease Genetics; DIAGRAMplusGERAplusUKB, DIAbetes Genetics Replication and Meta-analysis (DIAGRAM), Genetic Epidemiology Research on Aging (GERA) and the UK, Biobank (UKB).

**TABLE 5:** Mediation analysis of the mediation effect between RuminococcaceaeUCG010 and CAD/MI *via* T2DM.

Outcome		Mediator (T2DM)	Direct effect	Mediation effect	Proportion of mediation effect (100%)
CAD	FinGen	FinnGen	0.041	0.034	14.84
		DIAGRAMplusGERAplusUKB	−0.024	0.029	11.80
	CARDIoGRAMplusC4D	FinnGen	0.043	0.035	27.86
		DIAGRAMplusGERAplusUKB	−0.009	0.032	25.48
MI	FinGen	FinnGen	0.071	0.038	11.88
		DIAGRAMplusGERAplusUKB	0.062	0.037	11.57
	CARDIoGRAMplusC4D	FinnGen	−0.0003	0.029	22.31
		DIAGRAMplusGERAplusUKB	0.051	0.030	23.08

CAD, coronary artery disease; MI, myocardial infarction; T2DM, Type 2 diabetes; CARDIoGRAMplusC4D, Coronary Artery Disease Genome-Wide Replication and Meta-analysis plus the Coronary Artery Disease Genetics; DIAGRAMplusGERAplusUKB, DIAbetes Genetics Replication and Meta-analysis (DIAGRAM), Genetic Epidemiology Research on Aging (GERA) and the UK, Biobank (UKB).

## 4 Discussion

Metabolic risk factors such as diabetes, dyslipidaemia and obesity often converge and interact with each other to significantly increase the risk of cardiometabolic diseases, which is an important strategy for the primary prevention of CAD. The occurrence of one metabolic disease is associated with a significantly increased risk of the second. In a cohort of 0.5 million Chinese adults, the multimorbidity rate was 15.8%. The highest mortality is caused by cardiometabolic multimorbidity, and any combination of cardiometabolic multimorbidity has a multiplicative mortality risk (Fan et al., 2022). The gut–heart axis has been proposed on the basis of accumulating evidence that gut dysbiosis plays a crucial part in the progression of atherosclerosis. Consequently, the composition and function of the GM is a potential target to improve prognosis (Chen et al., 2020; Bianchi et al., 2022). Our study aimed to evaluate the causal effect between the GM and CAD/MI based on the current correlation study, and to provide a new metabolic intervention target for CAD.

In this study, we conducted MR analysis at different levels of GM bacterial taxa. At the phylum, class, order and species levels, no association between the GM taxa and CAD or MI risk was found in any database. However, the results suggested that increased abundance of the RuminococcusUCG010 genus reduced the risk of CAD and MI with consistent results in both meta-analysis and repeated analysis of the UKB dataset. No horizontal pleiotropy was detected in our study. In MVMR and mediator analysis, we further found that T2DM was involved as a mediator in the protective effect of RumicocusUCG010 on CAD/MI. Our findings provide novel ideas for future approaches to the prophylaxis and treatment of CAD or MI, that is, targeted modulation of dysbiosis of specific GM taxa.

The GM comprises several species of microorganisms, including bacteria, yeast, and viruses. Taxonomically, bacteria are classified by phyla, classes, orders, families, genera, and species. The dominant microbes of the GM belong to five phyla (Bacteroidetes, Firmicutes, Actinobacteria, Proteobacteria and Cerrucomicrobia), with Bacteroidetes and Firmicutes accounting for 90% (Arumugam et al., 2011). Ruminococcus (family Oscillospiraceae, phylum Firmicutes) is a gut-associated butyrate-producing bacterial genus. Butyrate, a short-chain fatty acid (SCFA), has powerful anti-inflammatory properties that inhibit proinflammatory responses by intestinal macrophages (Louis et al., 2007; Arpaia and Rudensky., 2014). Increased inflammation has been confirmed to play a role in the onset of atherosclerotic CVD. Experimentally induced acute endotoxemia can aggregate inflammatory factors and lead to vascular endothelial damage, while chronic endotoxemia is related to metabolic syndrome (MetS) (Boutagy et al., 2016). Kazuyuki and Kang et al. reported that butyrate and butyrate producing Ruminococcus can reduce the development of endotoxemia and atherosclerosis in mice (Kang et al., 2017; Kasahara et al., 2018). In a population cohort study, involving 161 CAD patients and 40 healthy controls, a co-abundance bacterial group containing Lachnospiraceae and Ruminococcaceae was negatively associated with CAD development (Liu et al., 2019). However, there is no significant causal effect between butyrate and CAD/MI in our study. It should be noted that PWY-5022 represents as a proxy for intestinal butyrate production, but not be directly related to the amount of butyrate absorbed by the host. The species of bacteria most associated with the abundance of PWY-5022 are Eubacterium rectale and Roseburia integralis, and the metabolic pathway correlated with RuminococcusUCG010 needs to be found to improve the reliability of the results. Arterial stiffness is known as an early manifestation of atherosclerosis, and carotid-femoral pulse velocity (cfPWV) is considered the gold standard for measuring arterial stiffness (Katsiki et al., 2016). A recent study involving 617 women from the TwinsUK cohort revealed a negative correlation between arterial stiffness and the abundance of the Ruminococcaceae family (Menni et al., 2018). As the primary risk factor for cardiovascular disease, diabetes has been an important target for the prevention and control of CAD. It has been found that daggligin and metformin produce hypoglycaemic effects by improving the structure of the microbiota. Ruminococcaceae was the dominant enterotype in two groups of medicated mice, which indicates that Ruminococcaceae plays a potential role in regulating blood glucose (Yang et al., 2020). Our study further confirmed the mediation effect of T2DM on the causal effect of RuminococcusUCG010 and CAD/MI that RuminococcusUCG010 can reduce the risk of CAD by reducing the incidence of T2DM. The above study findings were consistent with our conclusions. Interestingly, another study found that Ruminococcus was enriched in stable angioma (SA) and acute myocardial infarction (AMI) through faecal gene sequencing of 261 patients with CAD (Zhang et al., 2022). In fact, in human ecology, the GM exists as functional groups named ‘guilds’, with pivotal members of each guild growing or declining together in response to changes in the physiological environment. In clinical cohort studies, the composition of the GM also changes dynamically with long-term development of CAD (Faust and Raes., 2012). In conclusion, our data suggested that, overall, a high abundance of RuminococcusUCG110 has a long-term protective effect against CAD or MI, in which T2DM plays an important mediating effect.

The MR study design is a major merit of this study. The two-sample MR explored the linear relationship between exposure and outcome and MVMR and Mediation analysis was conducted to investigate a potential non-linear association. Genetic variation is a stable and measurable exposure factor that diminishes confounding factors and reverse causality and therefore reinforces causal inference. Based on an adequate sample size of data, stringent quality control conditions and analysis methods were adopted. We used multiple models to evaluate causal effects and conduct repeated validation in different databases. In addition, several sensitivity analyses were performed in our study. The research results were reliable and stable. Compared to a single exposure MR study, the exposure factor, namely, the GM, in this study is complex and massive with a huge workload and analysis challenges. There were several limitations to this study that need to be taken into account. First, two-sample MR requires two independent samples from the same ethnicity but without overlap. This study aimed to ensure that both databases were from European populations as much as possible to avoid ethnic bias. However, in the absence of individual data, the independence of the two samples cannot be strictly guaranteed. Second, the statistical power for the UK Biobank data analysis did not reach the 80% threshold, which may be caused by slight variance in the GM explained by the selected IVs. Third, this study focused on European populations, and subsequent comprehensive studies will need to be carried out across different ethnic groups.

## 5 Conclusion

This MR study provided suggestive genetic evidence that the higher the RuminococcusUCG010 abundance is, the lower the risk of CAD and MI, with T2DM playing a mediating effect. This genus may become a novel target in strategies for treating and preventing CAD and MI.

## Data Availability

The original contributions presented in the study are included in the article/Supplementary Material, further inquiries can be directed to the corresponding author.
